# How Do Consumers Perceive Sensory Attributes of Apple?

**DOI:** 10.3390/foods10112667

**Published:** 2021-11-02

**Authors:** Pakeza Drkenda, Asmira Ćulah, Nermina Spaho, Asima Akagić, Metka Hudina

**Affiliations:** 1 Faculty of Agriculture and Food Sciences, University of Sarajevo, Zmaja od Bosne 8, 71000 Sarajevo, Bosnia and Herzegovina; p.drkenda@ppf.unsa.ba (P.D.); culah.asmira@gmail.com (A.Ć.); n.spaho@ppf.unsa.ba (N.S.); a.akagic@ppf.unsa.ba (A.A.); 2 Department of Agronomy, Biotechnical Faculty, University of Ljubljana, Jamnikarjeva 101, SI-1000 Ljubljana, Slovenia

**Keywords:** consumer assessment, firmness, soluble solids content, titratable acidity, overall impression

## Abstract

Pomological characteristics and consumer acceptability of four scab-resistant apple cultivars (‘Topaz’, ‘Florina’, ‘Goldstar’ and ‘Golden Orange’) and standard commercial cultivar ‘Golden Delicious’ were investigated. Consumer acceptability consisted of rating fruit samples on Likert scales measuring appearance, flavour, size, sweetness, acidity, crispiness, juiciness, skin texture and general impression. Consumers better evaluated the cultivar ‘Topaz’ sensory characteristics of flavour, juiciness, taste and general impression than other evaluated scab-resistant apple cultivars and the cultivar ‘Golden Delicious’. ‘Golden Delicious’ got good grades for appearance, size and sweetness. ‘Topaz’ also had the best pomological characteristic related to measured fruit firmness, contents of soluble solids and organic acids. It can be concluded that only the cultivar ‘Topaz’ among the scab-resistant apple cultivars achieved a good consumer assessment.

## 1. Introduction

The ongoing trend of fruit production in the world and Europe is ecologically responsible farming systems (organic and integrated production), which imply minimal or no application of chemical plant protection products. In order to achieve economical production in such systems, farmers must grow varieties that are resistant to the most economically important diseases. The economically important diseases of apple are scab (*Venturia inaequalis* (Cooke) G. Wint./Aderh.) and powdery mildew (*Podosphaera leucotricha* (Ellis and Everh.) E. S. Salmon).

A large number of resistant apple varieties have been created through breeding work. Despite the large number of newly created apple cultivars, only twelve of them are represented in wide production practice [1].

Scab is one of the most important leukotic diseases in apple production. To overcome this disease, it is necessary to monitor the orchard regularly, and to apply fungicides frequently, which results in high production costs. According to Kühn and Thybo [2], resistant varieties have great ecological and economic advantages. However, the newly resistant varieties do not always have satisfactory fruit quality that is acceptable to consumers. It is therefore important to discover which properties are relevant to the consumer acceptability of apples. Sensory analysis is a valuable tool for assessing the market potential and success of resistant apple cultivars. The success of newly developed resistant apple cultivars depends on their fruit quality, which should be at least equal to that of commercial cultivars [3]. Kellerhals et al. [4] also emphasized this. Human perceptual abilities are important for a complete human life. Consumers often choose a specific apple cultivar based on their sensory characteristics. The requirements of consumers are changing and their expectations in terms of fruit quality are increasing [5,6,7]. Consumer taste is a dynamic category that results in the consumption of different cultivars. However, fruit quality is a complex category and depends on the pomological sensory properties of fruit (size, shape, skin colour, flavour, taste, juiciness, crispness, firmness etc.), and is affected by agricultural practices of cultivation and the nutritional richness of the fruit [8,9,10,11].

Many authors have done research related to sensory analysis and consumer preferences of different apple cultivars [6,9,12,13,14,15,16]. Although results on the sensory quality of scab-resistant apple cultivars can be found in the literature [2,3,12,17,18,19,20,21,22], no results have so far been presented from Bosnia and Herzegovina (B&H).

Fruit production in B&H has great potential, but is also limited by insufficient knowledge of suitable cultivars. Apple scab (*Venturia inaequalis* Cooke (Wint)) is the most damaging disease. The best way to avoid this problem is to grow scab-tolerant or resistant apple cultivars. Because of breeding programs, several scab-resistant cultivars are now available. However, Bosnian growers lack presence and knowledge about scab-resistant cultivars. The most popular and important apple cultivar is ‘Golden Delicious’, but this cultivar is susceptible to scab. The cultivars ‘Golden Orange’ and ‘Goldstar’ could be suitable as an alternative to ‘Golden Delicious’ for organic fruit production in B&H. There are new promising scab-resistant cultivars that fruit growers are still waiting for because they do not know enough about these cultivars and because they are afraid that consumers will not accept these varieties. 

On the other hand, the only known scab-resistant cultivar grown in modern B&H orchards is ‘Topaz’. Therefore, it is very important to increase the level of knowledge about new scab-resistant apple cultivars among apple producers and to improve consumer preference for scab-resistant apple cultivars. Variety selection remains one of the many challenges farmers face when managing an orchard. Growers must tailor cultivar selection to their target market.

The introduction and success of a new cultivar are determined by consumer preferences. However, this is influenced by various parameters [3,9,23,24]. It has been reported that consumer preferences can vary over geographical locations as well as between demographic groups within a population [6,19,25,26]. Taste, aroma, texture and appearance are considered to be the most important sensory attributes of fruits [27].

Consumer preferences and sensory evaluation of some selected apple cultivars (‘Golden Delicious’, ‘Florina’, ‘Topaz’ and ‘Goldstar’) have already been studied and the data have been published [2,28,29,30,31]. However, there are no accurate results on consumer preferences for the apple cultivar ‘Golden Orange’.

The main aim of this study was to evaluate and compare consumer acceptability and the most important pomological characteristics (fruit size, firmness, total soluble solids content and total titratable acids) of scab-resistant apple cultivars in comparison with the commercial cultivar ‘Golden Delicious’. Based on the results of this work, recommendations could be made to apple producers for the introduction of a wider range of scab-resistant cultivars into production. 

## 2. Materials and Methods

### 2.1. Material

The experimental design included five apple cultivars: four scab-resistant cultivars (‘Topaz’, ‘Florina’, ‘Goldstar’ and ‘Golden Orange’) and one standard apple commercial cultivar (‘Golden Delicious’ clone Reinders). The apples were grown in the Drina Valley, and harvested at optimal maturity. The cultivars were grafted onto rootstock ‘MM106’, planted at 4 × 2 m, and trained as a central leader. The apple trees were cultivated with minimum pesticide use, according to the integrated fruit production method [32]. 

A three-member expert team determined the optimal maturity stage for each of the investigated varieties based on maturity indicators (iodine starch test, total soluble solids content, fruit flesh firmness and titratable acid content) and the fruits were harvested at the commercial maturity stage.

### 2.2. Pomological and Main Physicochemical Analysis

At harvest, 150 fruits were selected based on uniformity and absence of damage. After harvest, the fruits were stored in a cold room at 2 °C (± 1 °C), at a relative humidity of 95%. Before the sensory and pomological analysis, fruit were removed from the cold room and left for 24 h at room temperature. Thirty fruits were then separated for laboratory analyses of pomological characteristics, such as fruit weight, fruit flesh firmness, total soluble solids content and titratable acid content.

Fruit flesh firmness was performed by digital penetrometer twice per fruit: on the sunny side and on the shaded side. Total soluble solids content was measured using an Atago digital refractometer and expressed as °Brix. Total titratable acidity was determined as the % of malic acid by titration of the juice with 0.1 M NaOH.

### 2.3. Consumer Test 

Consumer test sensory analysis of apples was performed with a sensory central location test (CLT) in the hypermarket ‘KONZUM’ in Sarajevo and an intercept procedure was used. A team of six students was in charge of presenting samples and keeping records. They instructed consumers on how to complete the questionnaire. Before the sensory evaluation of the apples, consumers were asked to fill in a questionnaire, which consisted of several demographic items related to apple consumption behaviour depending on gender and age. One hundred and five consumers (65 women and 40 men) tested the apples. Four age groups were defined: younger than 20 years (14 consumers), 21–42 years (31 consumers), 42–60 years (40 consumers) and over 60 years (16 consumers).

All samples of apples were presented as whole fruit and sliced in cups labelled with a three-digit blinding code. A Likert 5pt-scale (where 1 is dislike a lot and 5 is like a lot) was used. The experiment was set up according to a balanced block design using the Latin square method. Each consumer evaluated eleven sensory attributes: size, appearance, colour, firmness, skin texture, juiciness, sweetness, sourness, flavour, taste and overall (general) impression. 

### 2.4. Statistical Analysis

The trial had a completely randomised design and the data were analysed using the Statgraphics Plus 4.0 program (Manugistics, Inc.; Rockville, MD, USA). The data of the pomological analysis and sensory evaluation were tested for differences between the cultivars using one-way analysis of variance (ANOVA; general linear model). Differences between cultivars were tested with Tukey’s high significance difference (HSD) test at a 0.05 significant level. Means and standard deviation (mean ± Sd) are reported. For measuring the relation between each pair of attributes, chemical and sensory, the Pearson correlation coefficient was calculated. Principal Component Analysis (PCA) was used to treat sensory and pomological results.

## 3. Results and Discussion

### 3.1. Pomological and Physicochemical Parameters of Apples 

The results of the basic physicochemical parameters of apples are presented in Table 1.

As can be seen (Table 1), the apple cultivar had a significant impact on all measured pomological parameters of fruit quality, except for total soluble solids content. 

The fruit weight ranged from 149.43 g with ‘Golden Orange’ to 192.77 g with ‘Florina’. ‘Florina’ had a significantly higher fruit weight than the other apple cultivars.

The fruit weight of ‘Florina’ was higher than that reported by Lanauskas et al. [33] and Dan et al. [18], but lower than reported by Militaru et al. [21]. Dan et al. [18] and Militaru et al. [21] found a lower fruit weight of ‘Golden Delicious’. Militaru et al. [21] reported smaller fruits of ‘Goldstar’ apple. The fruit weight of ‘Topaz’ was higher than reported by Lanauskas et al. [33] and Militaru et al. [21], but lower than reported by Greene and Clements [34]. 

The results presented in Table 1 indicate that fruit flesh firmness ranged from 4.12 kg cm^−2^ to 6.21 kg cm^−2^. ‘Goldstar’ had the highest value of fruit firmness, compared to the values of the other studied apple cultivars. ‘Golden Delicious’ had significantly the lowest value of fruit flesh firmness (except in comparation with ‘Golden Orange’).

Péneau et al. [35], Dan et al. [18] and Bonany et al. [9] found even higher values of fruit flesh firmness of ‘Golden Delicious’. Militaru et al. [21] and Kühn and Thybo [2] found lower fruit flesh firmness of ‘Golden Delicious’ than achieved in presented experiment. Fruit flesh firmness of ‘Topaz’ was higher than reported by Militaru et al. [21] and Lanauskas et al. [33], but lower than reported by Péneau et al. [35]. Dan et al. [18] reported a higher value of fruit flesh firmness with ‘Florina’, but Lanauskas et al. [33] cited a lower value of fruit flesh firmness of the same cultivar. Fruit flesh firmness of ‘Goldstar’ was higher than that reported by Lanauskas et al. [33].

In terms of total soluble solids content, the highest value was reached by ‘Goldstar’ (14.33 °Brix), while ‘Topaz’ and ‘Florina’ had the lowest value of this parameter (12.33 ºBrix), but differences in total soluble solids content between cultivars were small and not significant (Table 1). Militaru et al. [21], Bonany et al. [9], Péneau et al. [35] and Greene and Clements [34] found a higher content of TSS in ‘Golden Delicious’ and ‘Topaz’. Kühn and Thybo [2] reported lower TSS content in ‘Golden Delicious’.

Titratable acidity (TA) was determined from 0.28 to 0.55%. ‘Topaz’ had a significantly higher TA than the other cultivars (except ‘Goldstar’). There was no significant difference in the content of total titratable acids among the other cultivars.

Péneau et al. [35], Kühn and Thybo [2], Bonany et al. [9] and Militaru et al. [21] found higher TA in ‘Golden Delicious’. Militaru et al. [21] reported a lower TA content in ‘Topaz’, but Péneau et al. [35] reported a higher content of TA in this cultivar. 

According to consumer tests, ‘Golden Delicious’ the acceptance threshold is 4.5 kg cm^−2^ for flesh firmness and 12 °Brix for sugar content [36]. According to our results, the sugar and flesh firmness values of ‘Golden Orange’ are closer to these values than the other two Golden cultivars (‘Golden Delicious’ and ‘Goldstar’). 

### 3.2. Sensory Attributes

The results of the consumer sensory test are presented in accordance with different sensory attributes in Table 2, Table 3 and Table 4. The results of external quality attributes of the tasted apples are shown in Table 2.

Sensory values of fruit size ranged from 4.18 to 4.70. ‘Golden Delicious’ had significantly better sensory evaluation of fruit size than its resistant clones (‘Goldstar’ and ‘Golden Orange’) and ‘Topaz’. There was no significant difference in fruit size between the resistant clones of ‘Golden Delicious’ (‘Golden Orange’ and ‘Goldstar’). These results disagree with the report of Dan et al. [18]. It was shown in that study that consumers better accepted medium-sized fruits than very large fruits. 

There were no significant differences between ‘Golden Orange’, ‘Goldstar’ and ‘Topaz’ in terms of fruit size. This means that ‘Golden Orange’ and ‘Goldstar’ have the same acceptance of fruit size as ‘Topaz’. 

The sensory values of fruit colour ranged from 3.98 to 4.73. The cultivars ‘Florina’ and ‘Topaz’ obtained significantly better sensory values for fruit colour than the other apple cultivars studied (Table 2). It can be concluded that red fruit colour (of ‘Topaz’ and ‘Florina’) is more accepted by consumers than yellow fruit colour. Significantly lower sensory values for fruit colour were obtained for ‘Goldstar’. 

Fruit colour is one of the important quality parameters for consumers in selecting their favourite apple fruit [32]. Jönsson and Nybom [19] in their research on the acceptance of resistant apple varieties found that an attractive colour (red) and a satisfactory sugar level is important for acceptance by Swedish consumers. Of the ‘Golden Delicious’ clones, ‘Goldstar’ had a significantly lower score for colour than the other ‘Golden Delicious’ clone (Table 2).

Sensory values of fruit appearance ranged from 4.24 to 4.66. ‘Golden Delicious’ and ‘Golden Orange’ had the best sensory evaluation for fruit appearance, significantly better than those of ‘Goldstar’ and ‘Topaz’ (Table 2). ‘Florina’ was significantly better evaluated than ‘Goldstar’, but not than ‘Topaz’. The appearance of ‘Golden Orange’ fruit is as acceptable to consumers as the appearance of ‘Golden Delicious’ fruit. Blažek et al. [29] reported that ‘Florina’ and ‘Golden Delicious’ achieved the same appearance rating (agrees with our results). Some available results confirm that ‘Golden Orange’ was well accepted by consumers [37]. 

The results for apple texture are shown in Table 3 and it can be seen the examined cultivars were different in terms of fruit texture.

For firmness, the mean values of the cultivars ranged from 4.08 to 4.42. ‘Florina’ had a significantly higher mean value for firmness than ‘Golden Delicious’. There were no significant differences in firmness between ‘Golden Delicious’ and its clones. The mean values of cultivars for skin texture ranged from 3.88 to 4.22 (Table 3). ‘Golden Orange’ had a higher value for skin texture than ‘Goldstar‘ but not higher than ‘Golden Delicious’. For juiciness, cultivar means ranged from 4.23 to 4.58 and there were no significant differences between cultivars. 

Values of internal quality attributes of apple are shown in Table 3. The average value for sweetness ranged between 3.53 and 4.42 (Table 4). ‘Golden Delicious’ had the highest value of sweetness, while ‘Florina’ had the lowest. There were no statistical differences among the cultivars ‘Golden Orange’, ‘Goldstar’ and ‘Topaz’. ‘Golden Delicious’ had better value of sweetness than its clones (‘Golden Orange’ and ‘Goldstar’).

Sourness of the different apple cultivars ranged from 2.97 to 4.03 (Table 4). ‘Topaz’ and ‘Golden Orange’ were evaluated as significantly sourer than the other cultivars. ‘Goldstar’ and ‘Florina’ had intermediate values and there were no statistical differences among them (Table 4). Among the Golden cultivars, the sourness of ‘Golden Orange’ was rated better than that of ‘Golden Delicious’ and ‘Goldstar’. Blažek et al. [29] found that ‘Topaz’ scored worse in sourness than ‘Golden Delicious’ and ‘Goldstar’. 

Cultivar means for flavour ranged between 3.66 and 4.30 (Table 4). ‘Topaz’ and ‘Golden Delicious’ had the highest means of flavour. There were significant differences in flavour between ‘Topaz’ and ‘Golden Orange’, ‘Goldstar’ and ‘Florina’ as well as between ‘Golden Delicious’ and ‘Goldstar’ (Table 4). Among the Golden cultivars the flavour of ‘Golden Delicious’ was rated better than the sour taste of ‘Goldstar’. 

**Figure 1:**
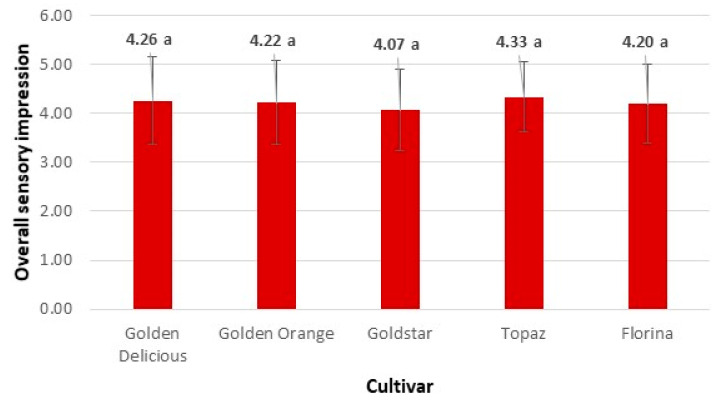
Mean value of overall sensory impression of the evaluated apple cultivars. The letter ‘a’ indicate that there is no statistically significant differences between cultivars at *p* ≤ 0.05.

The scores for taste of the different cultivars ranged from 4.13 to 4.36 (Table 4). There were no statistical differences in fruit taste between the apple cultivars studied. In the experiment by Blažek et al. [29], the taste of cultivar ‘Florina’ was worse than that of ‘Golden Delicious’. The final consumer evaluation of the tasted apples was overall impression. The results are given in Figure 1.

The overall impression of the different apple cultivars ranged from 4.07 to 4.33 (Figure 1). In terms of overall impression, there were no significant differences among the examined apple cultivars. All the apple varieties were equal good for the consumers. 

Blažek et al. [29] reported that ‘Florina’ had a worse taste than ‘Goldstar’. It should be noted that no apple cultivar had a score of less than four on six sensory attributes (overall impression, taste, size, appearance, firmness and juiciness). The greatest differences in sensory profiles were found in sourness (Table 4) and the least differences in overall impression (Figure 1).

Table 5 shows the correlation coefficients between overall impression and other sensory attributes (appearance, size, colour, firmness, skin texture, juiciness, sweetness, sourness and flavour).

There was a significant correlation between titratable acidity and the sensory attributes sweetness and sourness (*r* = −0.53, *p* = 0.05; *r* = 0.57, *p* = 0.05). 

It is interesting to note that the highest correlation was obtained between taste and overall impression (*r* = 0.65, *p* = 0.01). This agrees with a previous study of Jönsson and Nybom [19] who reported that taste is the most important factor for overall impression. 

Overall impression was also slightly correlated with juiciness (*r* = 0.51, *p* = 0.01) and firmness (*r* = 0.50, *p* = 0.01). There was no strong correlation between overall impression and other sensory attributes of the examined apple cultivars (Table 5). Dan et al. [18] reported that overall impression correlated only between juiciness and taste and, with some tolerance, with fruit size. The results of Péneau et al. [38] showed that freshness, together with taste and aroma, was a decisive attribute for selecting apples. Mieszczakowska-Frąc et al. [30] reported a strong correlation between overall quality and aroma (*r* = 0.545).

### 3.3. Consumer Profile Data

A total 105 consumers participated in the testing. There were 65 females (61%) and 40 males (39%). There were six consumers (5.7%) aged 10–20 years, 37 consumers (35.2%) in the age group 21–42 years, 50 consumers (47.6%) were 42–60 years old and 12 consumers (11.4%) were older than 60 years.

Overall impression for female consumers had the strongest correlation with taste, followed by juiciness, sweetness and flavour, whereas appearance, skin texture and firmness had no influence at all (Table 6). The overall impression among male consumers was positive in terms of firmness and skin texture. However, a negative correlation was found between these attributes in female consumers. 

For male consumers, taste was the most important factor for overall impression, followed by firmness, flavour and juiciness, whereas sourness had no influence at all. Flavour (followed by appearance and sourness) was the most important factor for overall impression for the youngest consumers. The other evaluated attributes had no influence on liking apple cultivars (Table 6). This result agrees with Dan at al. [18], who reported that flavour, together with taste, was the attribute that influenced consumers’ preferences the most, so it is important to be pleasant and balanced. For consumers between the ages of 21 and 42 years, overall impression significantly correlated with taste, followed by firmness, flavour and juiciness (Table 6). Overall impression for the consumers aged between 43 and 60 years had the strongest correlation with sourness, followed by juiciness, flavour and skin texture. Taste, followed by skin texture, firmness and juiciness significantly correlated with overall impression for the oldest consumers.

### 3.4. Principal Component Analysis

**Figure 2:**
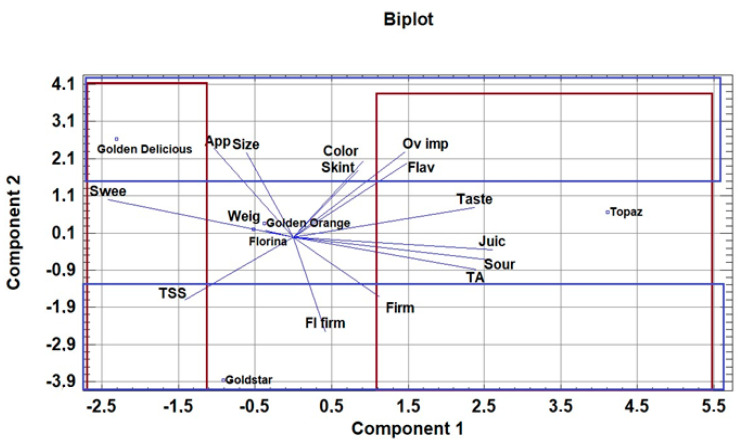
Principal component biplot of sensory and pomological attributes of different apple cultivars in the first and second component. Abbreviations: App—appearance; Flav—Flavour; Firm—Firmness; Fl firm—Fruit flesh firmness—kg cm^−2^; Juic—Juiciness; Ov imp—Overall impression; Sour—Sourness; Swee—Sweetness; TA—Total titratable acids—%, Skint—Skin texture; TSS—Total soluble solid contents—°Brix; Weig—Fruit weight—g.

Principal component analysis (PCA) was conducted to establish the relationships between sensory attributes, pomological and physicochemical parameters of the six tested apple cultivars (Figure 2). In accordance with the aim of this study, it is interesting to note the relation between ‘Golden Delicious’ and the four scab-resistant apple cultivars. In this PCA model, 78.56% of total data variance was explained by the two first components. Figure 2 shows the score and loading plots corresponding to component 1 vs. component 2 from the PCA model.

In the plot of sensory attributes, sourness, juiciness, taste, flavour, overall impression as well as the content of total titratable acids, are situated in the positive part of component 1. Sweetness and total soluble solids attributes are on the opposite, negative part of component 1. 

The second component in the negative part was significantly described by fruit flesh firmness, firmness and total soluble solids content. The external attributes, such as fruit size and appearance, followed by flavour, skin texture and overall impression, were situated in the positive part of component 2. ‘Golden Delicious’ is predominantly determined by fruit size, appearance, skin texture, colour, sweetness, flavour and overall impression. It means that consumers better accepted medium-sized fruits than very large fruits (Component 1).

The plot in Figure 2 clearly shows that ‘Golden Delicious’ and ‘Goldstar’ are located in the negative part of component 2. 

‘Goldstar’ is predominantly determined by firmness, fruit flesh firmness and total soluble solids content. These are the characteristics that distinguish ‘Goldstar’ from the other tested cultivars and, because of that, it was less acceptable for the consumers.

‘Topaz’ is the only cultivar located on the right side of the first component, diagonally opposite to ‘Golden Delicious’. It can be seen that ‘Topaz’ is determined mainly by attributes determined by component 1. These are the sensory attributes of taste, flavour, juiciness and sourness, but also the content of total titratable acids in the fruit. These parameters distinguish ‘Topaz’ from ‘Golden Delicious’. ‘Topaz’ was therefore the best evaluated cultivar by consumers, which is in accordance with other research that has evaluated this cultivar as well accepted. However, a common characteristic of ‘Topaz’ and ‘Golden Delicious’ is good overall impression. That means that these two varieties are generally well accepted. ‘Golden Delicious’ is better evaluated than ‘Topaz’ mainly in terms of external attributes (size and shape), but also in terms of the chemical sensory attribute of sweetness.

**Figure 3:**
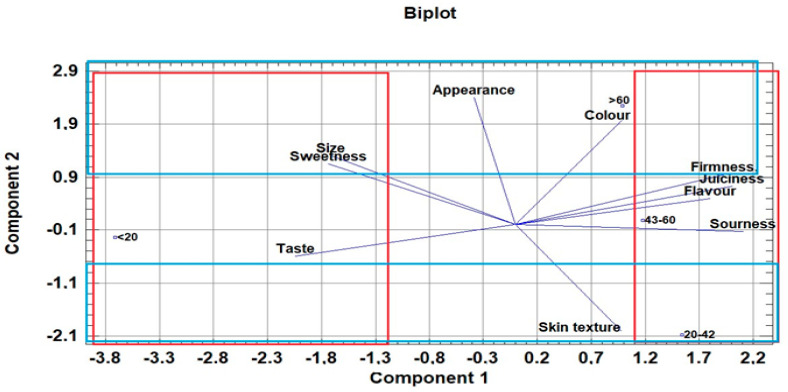
Principal component biplot of apple sensory attributes by different age group.

Other scab-resistant cultivars (‘Golden Orange’ as well as ‘Florina’) are located around the central zone of the biplot of Figure 2. This means that these cultivars are not affected by any of their characteristics, in either a positive or a negative sense. Principal component analysis (PCA) was also conducted to determine the relationships between sensory attributes and different age groups of consumers (Figure 3). 

For the middle-aged consumer groups (20–42 and 43–60 years), the most important sensory characteristics of apples are firmness, juiciness, flavour, sourness and skin texture (positive part of component 1 in Figure 3). However, it is noticeable that skin texture is particularly important for the 20–42 age group (negative part of component 2 in Figure 3). For the youngest age group, the most important attributes of apple cultivars were taste, size and sweetness (positive part of component 1 in Figure 3). The oldest age group defined colour, firmness, appearance, size and sweetness as the dominant attributes (positive part of component 2 in Figure 3). 

## 4. Conclusions

This study confirms that sensory analysis of consumer acceptance can be a good tool for the evaluation of apple cultivars. The results obtained confirm that it is possible to apply sensory analysis of consumer acceptance to understand the real impact of scab-resistant apple cultivars on consumer perception of apple fruit quality. In terms of many sensory attributes, the commercial cultivar ‘Golden Delicious’ is still more acceptable than the other scab-resistant apple cultivars (in size and sweetness better than both clones, in flavour and colour better than ‘Goldstar’). The only exception is ‘Topaz’. Among the scab-resistant apple cultivars, ‘Topaz’ scored the best consumer rating, followed by ‘Florina’. ‘Topaz’ received better scores than ‘Golden Delicious’ in terms of colour and sourness. Florina scored better than Golden Delicious in terms of colour. ‘Golden Orange’ scored better than ‘Golden Delicious’ in terms of skin texture. There were no significant differences in the attributes of juiciness and liking. The other examined scab-resistant clones of the cultivar ‘Golden Delicious’ require further breeding work in terms of pomological and sensory characteristics before consumers accept them. 

## Figures and Tables

**Table 1:** Average value of measured fruit weight, flesh firmness content and total soluble solids of the tested apple cultivars (mean ± standard deviation).

Cultivar	Fruit Weight (g) *	Fruit Flesh Firmness (kg·cm^−2^)	Total Soluble Solids (TSS, °Brix)	Titratable Acids (TA, %)
Golden Delicious	163.93 b ± 11.72	4.12 c ± 0.30	13.33 a ± 0.58	0.28 b ± 0.094
Golden Orange	149.43 b ± 9.18	4.34 c ± 0.28	12.83 a ± 1.89	0.33 b ± 0.041
Goldstar	160.88 b ± 12.78	6.21 a ± 0.44	14.33 a ± 0.58	0.42 ab ± 0.015
Topaz	160.39 b ± 18.18	4.99 b ± 0.32	12.33 a ± 2.08	0.55 a ± 0.015
Florina	192.77 a ± 18.52	5.05 b ± 0.33	12.33 a ± 1.52	0.32 b ± 0.054

* Different letters in columns ‘a–c’ indicate significantly different values between cultivars at *p* ≤ 0.05.

**Table 2:** Consumer evaluation of the fruit of five apple cultivars for external quality attributes—size, skin colour and shape (mean ± standard deviation).

Cultivar	Fruit Size *	Fruit Colour	Fruit Appearance
Golden Delicious	4. 70 a ± 0.62	4.49 b ± 0.71	4.66 a ± 0.59
Golden Orange	4.26 c ± 0.78	4.34 b ± 0.84	4.59 a ± 0.68
Goldstar	4.18 c ± 0.72	3.98 c ± 0.79	4.24 c ± 0.76
Topaz	4.41 bc ± 0.65	4.63 a ± 0.58	4.39 bc ± 0.76
Florina	4.52 ab ± 0.59	4.73 a ± 0.53	4.54 ab ± 0.71

* Different letters in columns ‘a–c’ indicate significantly different values between cultivars at *p* ≤ 0.05.

**Table 3:** Consumer evaluation of the fruit of five apple cultivars for texture quality attributes—firmness, skin texture and juiciness (mean ± standard deviation).

Cultivar	Firmness *	Skin Texture	Juiciness
Golden Delicious	4.08 b ± 0.952	4.10 ab ± 0.981	4.23 a ± 0.927
Golden Orange	4.38 ab ± 0.850	4.22 a ± 0.903	4.34 a ± 0.855
Goldstar	4.37 ab ± 0.764	3.88 b ± 0.942	4.35 a ± 0.890
Topaz	4.35 ab ± 0.798	4.15 ab ± 0.822	4.58 a ± 0.720
Florina	4.42 a ± 0.746	3.93 ab ± 1.082	4.37 a ± 0.825

* Different letters in columns a, b indicate significantly different values between cultivars at *p* ≤ 0.05.

**Table 4:** Consumer evaluation of the fruit of five apple cultivars for internal quality attributes—sweetness, sourness, flavour and taste (mean ± standard deviation).

Cultivar	Sweetness *	Sourness	Flavour	Taste
Golden Delicious	4.42 a ± 0.706	2.97 c ± 1.390	4.13 ab ± 1.124	4.17 a ± 0.950
Golden Orange	4.09 b ± 1.016	3.51 a ± 1.393	3.85 bc ± 1.205	4.15 a ± 0.911
Goldstar	3.92 b ± 0.921	3.44 bc ± 1.261	3.66 c ± 1.187	4.13 a ± 0.976
Topaz	3.53 b ± 1.284	4.03 a ± 1.194	4.30 a ± 0.944	4.36 a ± 0.723
Florina	3.95 c ± 0.969	3.35 bc ± 1.237	3.82 bc ± 1.130	4.13 a ± 0.832

* Different letters in columns ‘a–c’ indicate significantly different values between cultivars at *p* ≤ 0.05.

**Table 5:** Pearson correlation coefficients between sensory fruit attributes (appearance, size, colour, firmness, skin texture, juiciness, sweetness, sourness and flavour) and pomological fruit characteristics (weight, flesh firmness, total soluble solids and titratable acids).

	Size	Colour	Appearance	Skin Texture	Firmness	Juiciness	Sweetness	Sourness	Taste	Flavour	Overal Impression	Weight (g)	Firmness (kg cm^−2^)	TSS (°Brix)	TA (%)
Size	1	0.47 **	0.43 **	0.15 **	0.16 **	0.14 **	0.19 **	0.03	0.18 **	0.13 **	0.30 **	0.11	−0.10	−0.09	−0.34
Colour		1	0.52 **	0.21 **	0.25 **	0.22 **	0.16 **	0.14 **	0.27 **	0.30 **	0.37 **	0.16	0.06	0.26	0.16
Appearance			1	0.29 **	0.31 **	0.25 **	0.23 **	0.13 **	0.24 **	0.25 **	0.38 **	0.08	−0.11	0.64	−0.22
Skin texture				1	0.44 **	0.44 **	0.29 **	0.18 **	0.46 **	0.41 **	0.43 **	0.09	−0.08	0.07	−0.05
Firmness					1	0.60 **	0.25 **	0.34 **	0.54 **	0.35 **	0.50 **	0.18	0.10	−0.02	−0.18
Juiciness						1	0.31 **	0.31 **	0.57 **	0.40 **	0.51 **	0.22	0.03	−0.21	−0.03
Sweetnes							1	0.07	0.46 **	0.26 **	0.41 **	−0.02	−0.19	0.32	−0.53 *
Sourness								1	0.38 **	0.26 **	0.28 **	−0.16	0.22	0.21	0.57 *
Taste									1	0.51 **	0.65 **	−0.01	0.25	−0.13	0.42
Flavour										1	0.49 *	0.09	0.04	−0.34	0.25
Overal impression											1	0.10	−0.18	0.17	0.16
Weight (g)												1	0.02	−0.32	−0.50
Firmness (kg cm^−2^)													1	0.33	0.45
TSS (ºBrix)														1	−0.05
TA (%)															1

* Correlation is significant at a 0.05 level (2-tailed). ** Correlation is significant at a 0.01 level (2-tailed). TSS, total soluble solids; TA, tittratable acids.

**Table 6:** Pearson correlation coefficients between overall impression and other sensory attributes (appearance, size, colour, firmness, skin texture, juiciness, sweetness, sourness and flavour) by different gender and demographic groups.

Overall Impression	Size	Colour	Appearance	Skin Texture	Firmness	Juiciness	Sweetness	Sourness	Taste	Flavour
1. Gender groups										
Men	0.39 **	0.42 *	0.43 **	0.44 **	0.57 **	0.47 **	0.26 **	0.126	0.60 *	0.48 **
Women	0.24 **	0.32 **	0.02	−0.09	−0.10	0.53 **	0.49 **	0.35 **	0.66 **	0.49 **
2. Age groups										
Younger than 20	0.18	0.36	0.54 **	−0.05	0.10	0.03	0.09	0.43*	0.27	0.61 **
21–42 years	0.20 **	0.37 **	0.27 **	0.31 **	0.55 **	0.47 **	0.38 **	0.29 **	0.66 **	0.50 **
43–60 years	0.36 **	0.39 **	0.43 **	0.50 **	0.49 **	0.57 **	0.45 **	0.63 **	0.29 **	0.50 **
Over 60 years	0.39 **	0.32 *	0.43 **	0.60 **	0.59 **	0.58 **	0.42 **	0.28 *	0.85 **	0.43**

* Correlation is significant at a 0.05 level (2-tailed); ** Correlation is significant at the 0.01 level (2-tailed).

## Data Availability

Data are available at corresponding author on reasonable request.
